# How Human Medicines Are Disrupting Aquatic Ecosystems

**DOI:** 10.1021/acscentsci.5c01661

**Published:** 2025-09-09

**Authors:** Katarina Zimmer

## Abstract

More drugs are entering aquatic habitats. Scientists are teasing apart how they influence the behavior, reproduction, and biology of organisms that live there.

## Graphical abstract

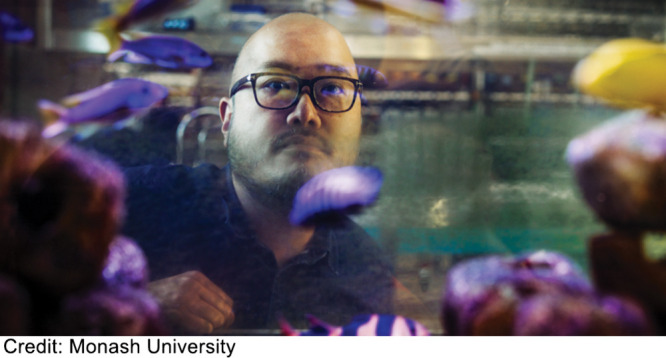

In 2020 and 2021, the Atlantic salmon swimming along Sweden’s River Dal were joined by some unusual newcomers. A team of scientists had introduced 279 young salmon, or smolts, into the river. Three-quarters of them carried implants that released pharmaceutical compounds into their bodies—either the seizure-treating benzodiazepine clobazam, the opioid pain-medication tramadol, or a mixture of both.

**Figure:**
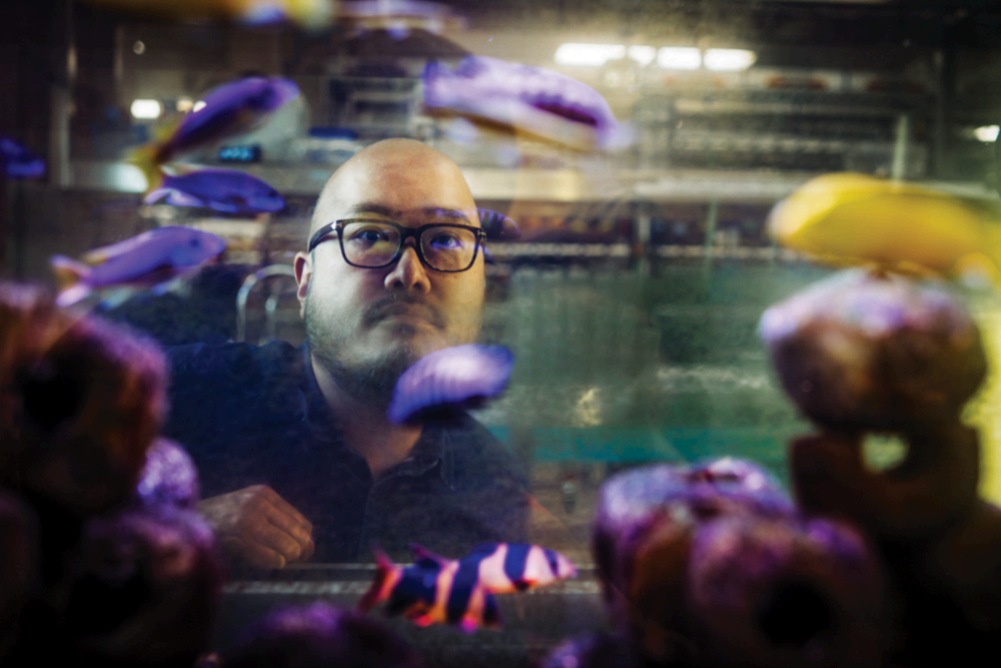
Ecotoxicologist Bob Wong studies how fish change when exposed to the antidepressant medication fluoxetine. Credit: Monash University.

As the young fish migrated down the 28 km stretch of river, past lurking predators and through rapids and dangerous hydropower dams, the scientists tracked their progress through telemetry implants. They wondered how many drug-exposed fish would survive this perilous journey and reach the ocean.

“Can pharmaceuticals alter animal behavior...in the wild?” Michael Bertram, a behavioral ecologist and ecotoxicologist at the Swedish University of Agricultural Sciences and the senior author of the study, wanted to know.

Around the world, growing quantities of unmetabolized drugs are excreted by human bodies and slip, along with wastewater, into lakes and rivers. Some pharmaceutical pollutants also stem from veterinary drugs used for livestock, come directly from pharmaceutical manufacturing areas, or occur through the improper disposal of unused or expired medicines. Historically, much early research aiming to discern their impact has relied on laboratory tests that expose organisms to unrealistically high concentrations of drugs, often finding outsized—and sometimes lethal—effects. But as studies like Bertram’s illustrate, the field is increasingly shifting to more environmentally relevant ways of studying pharmaceuticals.

Scientists are learning that even at low, environmentally realistic concentrations, drugs can have diverse—and often surprising—effects. Bertram and his colleagues, for instance, observed that twice as many clobazam-exposed salmon reached the Baltic Sea compared with fish that carried an empty implant. While that may bode well for individual fish, Bertram cautions that it could upset the delicate balance of their ecosystems as a whole. And studies on other drugs, like hormones or antidepressants, point to disruptive effects on wildlife behavior, development, and reproduction—motivating scientists to also explore ways of reducing pharmaceutical pollution.

“The key concern is that we have sublethal concentrations of chemicals in the environment that are having hidden effects,” Bertram says.

## How pharmaceuticals affect fish

Pharmaceutical pollution has been documented at least since the 1980s, but only recently have scientists begun to grasp the scale of the problem on a global level, thanks to faster and better water-analysis methods. In 2022 ecotoxicologist Alistair Bruce Alleyne Boxall of the University of York and his colleagues published a landmark survey on water samples collected from 1,052 sites in 104 countries and spanning 258 of the world’s rivers. Fifty-three of 61 assessed pharmaceutical compounds were widespread across the samples, from epilepsy and diabetes treatments to antidepressants, antibiotics, and benzodiazepines, many of which persist for long periods in the environment, Boxall says. Their concentrations were highest in low-to-middle-income countries, which often have limited wastewater treatment.

Scientists have documented a growing breadth of chemicals over time, which Boxall suspects will continue to increase as dozens of new compounds are being introduced to markets every year. Experts also expect the overall concentrations of such compounds to increase over the coming decades as medicine demand rises along with an aging global population. “If we carry on as we are,” Boxall says, “then the levels of pharmaceuticals are likely to go up.”

The good news is that many of the most severe effects of pharmaceuticals that have been documented in lab studies—death, deformation, and other maladies—generally occur only at doses that aquatic organisms would not normally encounter, notes ecotoxicologist Dalma Martinović-Weigelt of the University of St. Thomas, Minnesota. After all, “Pharmaceuticals have been carefully designed not to outright kill us.”

But more ecologically realistic studies are uncovering other effects on aquatic life. The implants used for Bertram’s salmon were designed to release 50 μg of pharmaceuticals per gram of implant material into each fish over the duration of their migration. The team estimated that the quantity absorbed by each fish would mimic exposures that have been observed in contaminated ecosystems elsewhere, says behavioral ecologist Jack Brand of the Swedish University of Agricultural Sciences, who led the study. The team had initially expected the greatest effects to come from the combination of tramadol and clobazam, as these exhibit dangerous interactions in people, but postmortem analyses of mixture-exposed fish suggested that their brains took up less of each chemical than fish exposed to each drug in isolation.

**Figure:**
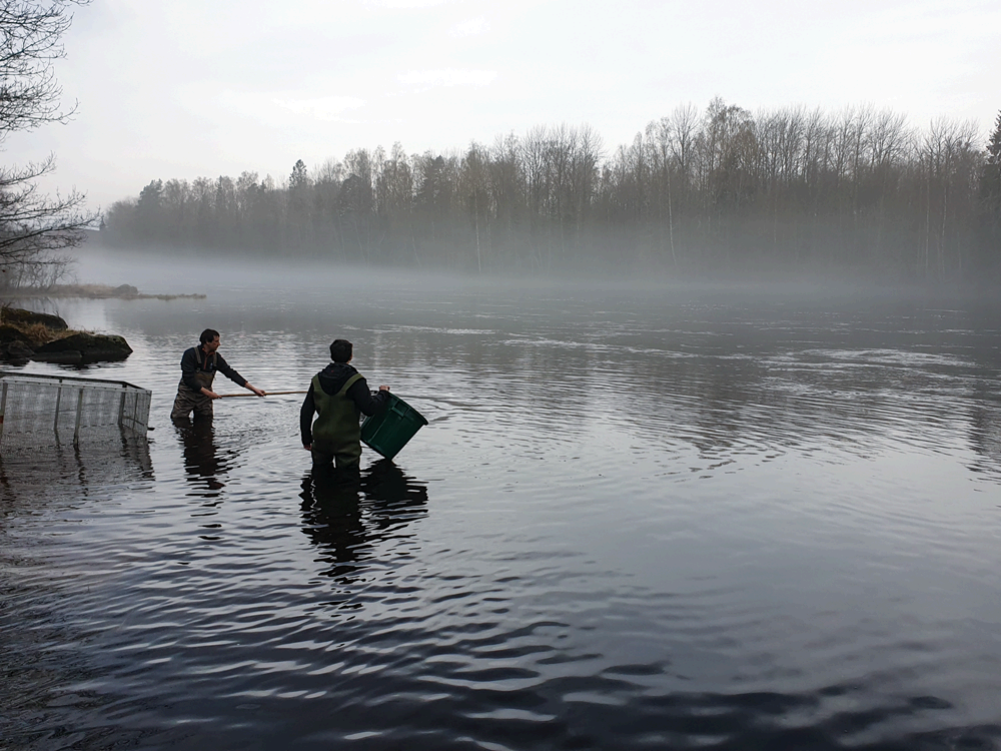
In 2020 and 2021, researchers released hundreds of drug-exposed salmon into Sweden’s River Dal to see how exposure to pharmaceuticals would affect their migration to the ocean. Credit: Michael Bertram.

The fish implanted with only clobazam experienced a more pronounced effect. Their improved survival was “not what we expected,” Bertram says. In previous studies where Bertram’s team spiked lakes with environmentally realistic levels of the benzodiazepine temazepam, the action decreased the migration success of brown trout, which swim up rivers to spawn. And when the team exposed six salmon smolts to clobazam in tanks, the fish became easier for predators to catch: groups broke apart rather than sticking together in safety. Bertram hypothesizes that clobazam, which reduces anxiety in humans by interacting with γ-aminobutyric acid type A (GABA_A_) receptors, has a similar effect in fish, whose brains also express this receptor—causing them to be less cautious around predators.

Bertram speculates that, in lakes, this reduced anxiety may make individual fish more likely to wander into contact with stealthy predators like northern pike. But in a river, a fish with reduced anxiety might simply go with the flow out to sea, slipping past predators unnoticed. It’s also possible that predators might be confused by their prey’s unusual behavior, as they usually screen for groups of salmon rather than individual swimmers. “The predators aren’t looking for those types of salmon smolts that are behaving strangely, and so [these] have, as a result, an increased migration success,” Bertram hypothesizes.

Other scientists, too, are realizing just how nuanced the effects of a single drug can be. Behavioral ecotoxicologist Bob Wong of Monash University in Australia and his colleagues have been collecting lake-dwelling guppies from the wild, putting them into large metal pools, and exposing them to environmentally relevant levels of fluoxetine, an antidepressant marketed as Prozac in the US. The effects are diverse, affecting everything from body condition to mating behavior, but they depend heavily on the individual animal, its sex, and the dose. In one study, for instance, fluoxetine increased the size of male guppies’ sperm-transfer organ and reduced the movement of their sperm—but only at low drug concentrations. “Sometimes you find disturbances at the lower concentrations but not at the higher concentrations,” Wong says.

The effects also depend on the duration of exposure. After being exposed to fluoxetine for a month, guppies become less likely to become prey, exhibiting a kind of protective freezing behavior after experiencing a simulated strike by a bird predator. But this effect disappeared after 8 months, when fluoxetine-exposed individuals behaved no differently than unexposed fish. “Maybe the fish are getting used to the toxicants in the environment,” Wong says. In principle, that could be a positive thing, as it reduces disruptions to ecosystems as a whole.

Other effects become apparent only after long-term exposures. In one 2-year study, fluoxetine-exposed guppies started to behave more similarly to one another—a big difference from unexposed fish, which exhibit a variety of shy and bold behaviors. This similarity is a problem because behavioral variation helps fish populations weather environmental changes—like a boon in predator populations—as it ensures that some well-equipped individuals are always present, Wong says. The results underscore just how complex the impacts of pharmaceuticals can be.

**Figure:**
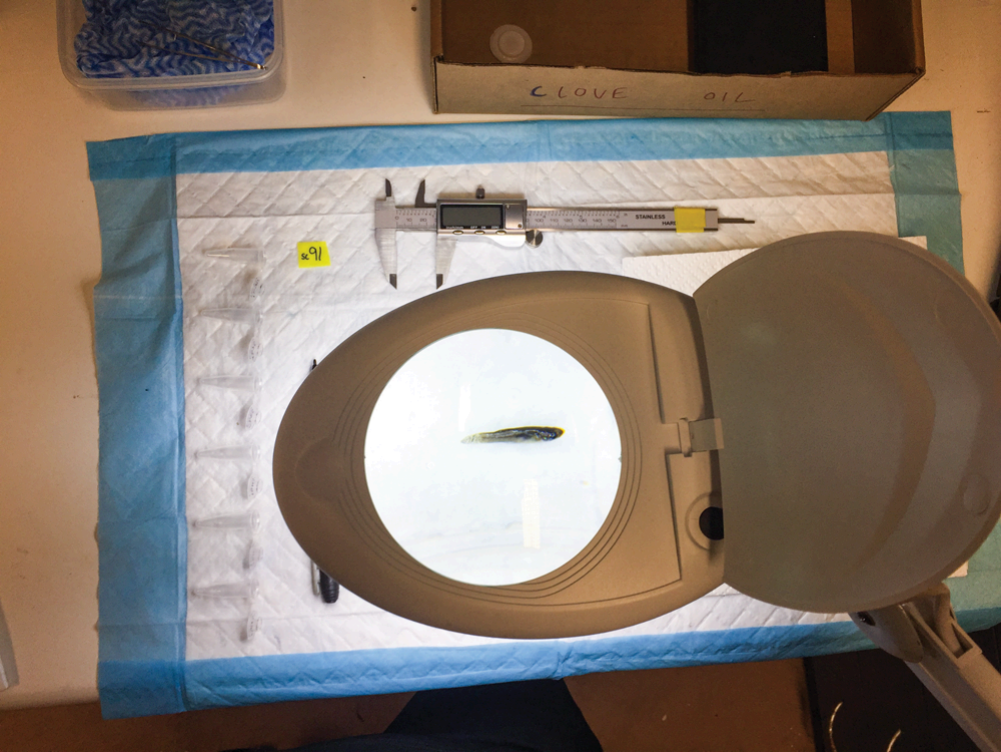
Bob Wong’s team measured mosquitofish as part of a project to see how drugs like fluoxetine affect fish biology and physiology. Credit: Jake Martin.

“Tests for determining whether a chemical poses a threat or not need to more carefully consider this complexity to avoid over- or underestimating the dangers for the environment,” he says.

## Diving into the mechanisms behind drug effects

It is a mystery why fluoxetine and other drugs have such nuanced effects—or how they even act in fish bodies. In general, fish and humans might respond similarly to drugs because many of the receptors that the drugs target—and the corresponding biological pathways—are evolutionarily conserved. Fluoxetine, for instance, might affect animals’ anxiety and boldness by boosting levels of serotonin, as it does in people, which influences the stress response.

But while fish and humans share many biological pathways, notable differences exist in physiology. These differences can cause drugs to have different effects on fish bodies than on humans. For instance, while painkillers like ibuprofen are mostly associated with relieving pain associated with prostaglandins (hormone-like substances) in people, they have a greater range of effects in fish, where prostaglandins also act as pheromones that play key roles in reproductive behavior, Martinović-Weigelt says. And distantly related organisms like invertebrates or plants may have even less-predictable responses; some studies show that fluoxetine, for instance, can hamper the reproduction of mussels and decrease the growth of algae.

“We’re constantly being surprised at how organisms respond to these compounds because often we don’t understand the physiology of all of these different species well enough,” says ecotoxicologist Karen Kidd of McMaster University.

Some scientists are turning to state-of-the-art tools to decipher these mechanisms. Ecotoxicologist Charles Tyler of the University of Exeter has long studied synthetic estrogens, which are used as hormone-based contraceptives and were one of the first pharmaceutical hormones found to have adverse effects on fish even at low concentrations. Research by Tyler and colleagues has shown that male fish exposed to estrogen-laced wastewater effluents produce eggs in their testes, and Kidd’s studies demonstrate that this feminization can cause populations to crash.

To understand how else estrogens may play a role in fish development, Tyler’s team spent several years developing transgenic fish that carry a gene engineered to express green fluorescent protein once estrogens bind to the corresponding receptor. In a 2022 study, they observed that, in developing zebrafish, the very first cells to respond to estrogen are in the smell-processing olfactory bulb. Estrogen, the results suggest, is necessary for the correct development of a fish’s sense of smell. Further experiments confirmed this result; blocking the estrogen receptor caused fish to lose their ability to smell food or sexual hormones.

**Figure:**
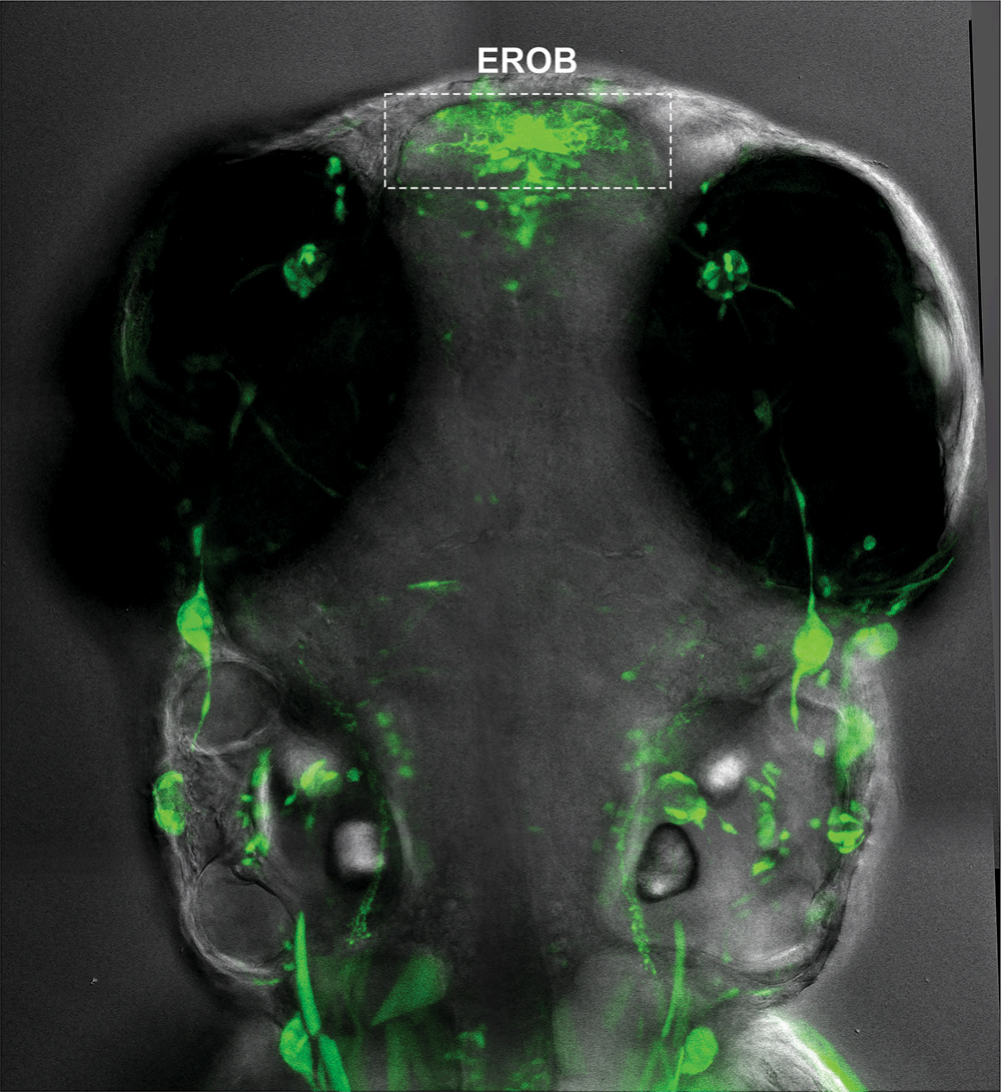
Ecotoxicologist Charles Tyler’s team designed transgenic zebrafish to express fluorescent protein once estrogen binds to its receptor. This experiment showed that the first cells to respond to estrogen during development are in the smell-processing olfactory bulb (called the estrogen-responsive olfactory bulb, or EROB). Credit: Aya Takesono.

Curiously, additional research hinted that exposing embryos to too much estrogen during early development has a similarly disruptive effect—suggesting that pollutant-exposed fish in the wild might have altered senses, affecting their survival, behavior, and ecology. “The consequences could be quite significant,” Tyler says.

### Preventing ripple effects on people and the environment

Scientists are starting to investigate other unanswered questions, like how drug exposures affect a population’s resilience, an organism’s offspring, or other parts of the food chain. The biggest research gap that experts are trying to fill is how different pharmaceutical compounds act in concert with one another. While most research has focused on studying individual compounds, aquatic organisms are exposed to a cocktail of many—and that means a cocktail of effects, says Erin S. McCallum, an aquatic ecologist at the Swedish University of Agricultural Sciences. Sometimes drugs may amplify each other’s effects, but other times they may not, as Bertram found with clobazam and tramadol. “The field is still trying to do a lot of research on how we can deal with mixtures of pharmaceuticals in the environment,” McCallum says.

Meanwhile, scientists are also thinking about ways to reduce pharmaceutical pollution. The challenge is that conventional wastewater treatment techniques—involving mechanical removal of solids and fats and employing microorganisms to absorb some polluting chemicals—don’t specifically target pharmaceuticals. Some countries, like Canada or Switzerland, use advanced wastewater treatments that employ techniques like chlorination, ozonation, and ultraviolet light, for instance, to kill off pathogenic microorganisms, which helps break down some pharmaceuticals. The European Union will soon require wastewater treatment plants to incorporate technology that can remove certain pollutants, with the pharmaceutical and cosmetics industries required to pay the bulk of the cost.

While the European Federation of Pharmaceutical Industries and Associations (EFPIA), a trade association for pharmaceutical companies, supports the polluter-pays principle, EFPIA is pushing back on the legislation in its current form; it’s unfair to hold only two industries accountable when a number of other sectors also contribute to wastewater pollution, according to spokesperson Kirsty Reid, EFPIA’s director for science policy.

Many experts don’t see advanced wastewater treatment technologies as a complete solution. Many low-to-middle-income regions lack even basic wastewater treatment, and even in developed countries, storms and floods cause untreated wastewater to be discharged. And the removal techniques aren’t fully effective for all compounds, says chemist Klaus Kümmerer of Leuphana University Lunenburg.

For some pharmaceuticals, “each of these new approaches can only remove, more or less, 30 to 50% of the parent compound,” he says.

He and others are working on developing more sustainable drugs that break down easily once in the environment. They recently succeeded in creating more sustainable variations of certain antibiotics by introducing a moiety that can be readily broken down under pH conditions of the bladder or sewage treatment plants. It is not about banning existing compounds, he says; it is about factoring in sustainability during the development of new ones.

**Figure:**
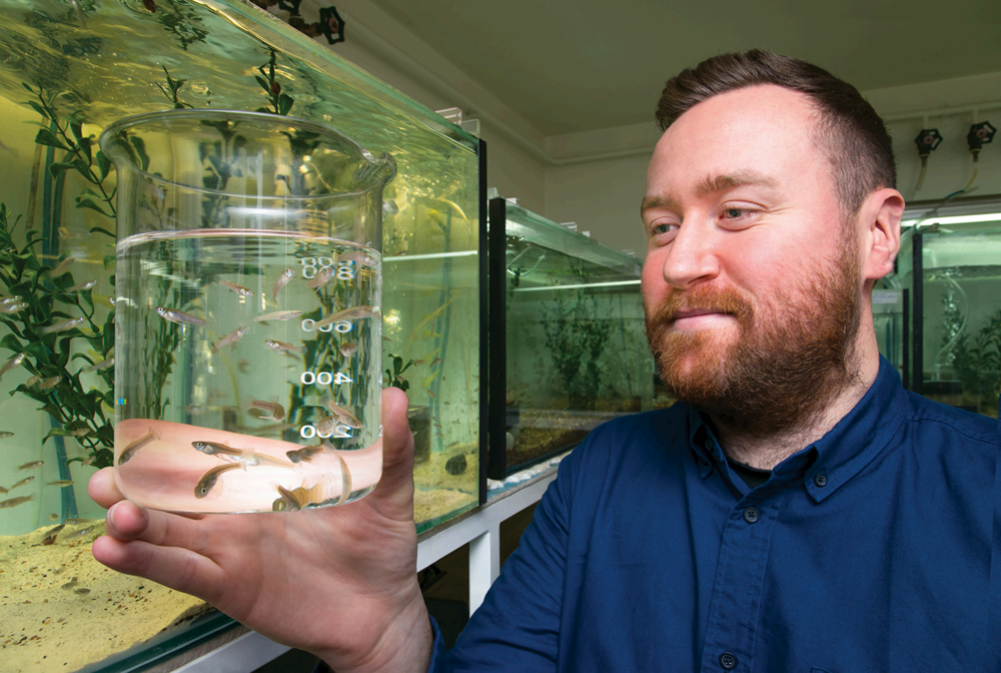
Michael Bertram, shown here holding a glass full of mosquitofish, studies the effects of pharmaceuticals on fish behavior. Credit: Steve Morton.

Such benign-by-design drugs could also be encouraged by policies that increase the importance of environmental risk assessments during the marketing authorization process, adds environmental law expert Mirella Miettinen of the University of Eastern Finland. But, she acknowledges, “it takes years to change the thinking and the processes.”

Creative solutions such as sustainable drugs may be necessary to prevent further disruption to ecosystems that are already suffering from other human-made problems, Bertram says. Regardless of whether effects may be positive or negative for individual fish, he suspects that drug-triggered changes have disruptive effects on ecosystems as a whole. The greater survival of clobazam-exposed Atlantic salmon, for instance, could cause dents in the populations of their invertebrate prey. Clobazam might also affect the timing of migration, causing salmon to arrive in the ocean too soon, when prey or temperature conditions aren’t right.

“Nature is extremely complicated,” Bertram says. “If we have these unseen agents of change that are pushing these populations one way or another, then that will have all sorts of effects on that population and the surrounding community.”

*Katarina Zimmer is a freelance contributor to* Chemical & Engineering News, *an independent news publication of the American Chemical Society.*

